# Charity Preachers

**Published:** 1873-03

**Authors:** 


### CHARITY PREACHERS.

POOR young men are every year provided with the money and clothing necessary to defray all their expenses while getting a ministerial education, as the State supports approved youths while they are preparing themselves for the army and the navy. In the abstract this is right and proper. If a man or woman chooses to support a theological student at the seminary, no one has any right to object to it; some of these "beneficiaries," as they are called, have been among the ablest and most useful men in the church. But the system is liable to abuse, and there is reason to believe that in the long run the church would be a gainer if no man was allowed to enter the ministry who had not the ability to make his way there by the strength of his own right arm, receiving no other aid than tuition free, absolutely free of all expense. Help is demoralizing, the world over, even parental help; self-help carries with it a momentum all through life, which helps to success in every undertaking; it gives courage to meet, and power to trample down obstacles, which many a time would appall one habituated to helping hands.

It is said that time is saved, but the Son of man was thirty years old before he entered the ministry; until then he worked as a carpenter, giving him bodily vigor, not hindering the mind and affections meanwhile from due preparation for the great work before him. Patrick Henry was the Demosthenes of revolutionary times; the most valuable period of his "preparatory" was as the proprietor of a country store, selling goods, weighing out tea and sugar and coffee, whittling sticks, talking politics, and telling jokes around the stove of a winter day; learning more and more of human nature, its weak points and its strong, and how to get at the feelings of men.

Abraham Lincoln's college was the flat boat, the "worm fence" and the wood pile, with their varied associations. It is the coming in contact with difficulties and discouragements which best elicits the moral force of a man. Every additional battle of the young soldier helps him that much more to fight better the next time; and in due course he becomes a veteran.

With advancing general intelligence and increase in the number of men of culture, the church needs more and more imperatively that its standard-bearers should be staid men—men of force, of courage, of self-assertion—of men who are more fully persuaded in their own minds, that their mission is true and that they stand on impregnable ground. Hence they should have full time to study the situation. Hence, there is such a thing as getting into the ministry too soon, before the judgment is matured, before a sufficient insight into human nature has been obtained.

Great discredit has often been brought on the church by the injudiciousness, the over zeal, the impatience, and the unwariness of young ministers, not indeed from any inherent viciousness, but from want of discretion and from inexperience of the wiles of the wicked.

Contact with the world as it is, is really as necessary a part of a theological education as the instructions of the "seminary." No man can get a truthful insight into human nature without having pecuniary transactions with his fellows, as in buying and bargaining, and selling and trusting, and being trusted. It is a miracle of grace if any man enters the ministry early, that he does not trip now and then, and come nigh to falling. If a young man works his own way into the ministry, he is not likely to marry too young; is more likely to marry a woman who will be a suitable help to him, and thus stand a chance of doubling his influence as a minister, instead of having that influence more than counteracted by an unfortunate match, or if not counteracted outright, he has a weight hanging on to him, pulling him downwards all the time. In the ministry, more than in any other profession, is it indispensable that the wife should be a co-laborer, not from the mere dictate of the brain, nor from a cold sense of duty, but from a genuine love of the Master and his cause. For a clergyman to have a wife who is a helpmeet suitable for him, is one of the greatest of blessings, and it is fair to presume that if a young man reaches thirty before he gets into the ministry he will be more likely to make a selection with such views, and less likely to be carried away with a pretty face, or a neatly turned ankle, or glossy curl. The bold fact stares us in the face everywhere, in all departments of learning and of trade, that the largest number of successful men are those who have made their own way to the summit, beginning at the very bottom rounds of the ladder, and without a penny to start with.

That most excellent paper, the *Scientific American*, narrates a case in point, that Samuel Lee, Professor of Hebrew, at the University of Cambridge, England, was seventeen years old before he ever thought of learning a foreign language. But every week he was able to save a few cents over his scant wages as a journeyman carpenter; with this he bought a book, read it, exchanged it for another. He was no eight-hour man, he did not want his employers to pay him for taking holiday, he was no clamorer for relaxation, never asked for help, but worked his full hours out and went direct to study, the very way to acquire bodily strength and mental vigor, exercising muscle and brain alternately; relaxation under such circumstances is a positive waste of time. For the twelve years, beginning with his nineteenth, he went to bed regularly at ten o'clock and went to work bright and early, yet from quitting work at sundown until bed time, he found leisure in these twelve years to learn and to be able to teach seventeen languages. And the *Scientific* truly remarks, in this connection, that where there is a will there is a way. If a young man wants to get into the ministry, and is fit to be there, he will hew a road for himself into it and ask favors of no one. Six hours a day is enough for any man to engage in hard study; it is as long as any young person ought to study; he can study to better advantage, and can learn more in six hours than in a greater number, while if he engages in bodily labor ten hours more, thus, resting the brain, and while he is studying, resting the muscles, he gives both all the recreation they need, and enters active or professional life with a vigorous mind in a healthy body, and not a poor consumptive looking, pale-faced, dyspeptic shadow of a man, who fairly puffs in ascending the pulpit stairs, and where a smelling bottle is as essential as his Bible. The moral of it is this: "Help yourself into your profession, if you want to feel yourself a man;" and then, wherever you may be placed, you will feel yourself master of the situation.

